# Short-Term Perceptual Training Modulates Neural Responses to Deepfake Speech But Does Not Improve Behavioral Discrimination

**DOI:** 10.1523/ENEURO.0300-25.2026

**Published:** 2026-03-13

**Authors:** Jinghan Yang, Haoran Jiang, Yanru Bai, Guangjian Ni, Xiangbin Teng

**Affiliations:** ^1^Academy of Medical Engineering and Translational Medicine, Tianjin University, Tianjin 300072, China; ^2^State Key Laboratory of Advanced Medical Materials and Devices, Tianjin University, Tianjin 300072, China; ^3^Haihe Laboratory of Brain-Computer Interaction and Human-Machine Integration, Tianjin 300392, China; ^4^Department of Psychology, The Chinese University of Hong Kong, Shatin, Hong Kong SAR 999077, China; ^5^Brain and Mind Institute, The Chinese University of Hong Kong, Shatin, Hong Kong SAR 999077, China

**Keywords:** deepfake perception, electroencephalography (EEG), learning and memory, perceptual learning, synthetic voices, voice recognition

## Abstract

Rapid advancements in artificial intelligence (AI) have enabled text-to-speech (TTS) systems to produce voices increasingly indistinguishable from humans, posing significant societal risks, particularly through potential misuse in fraud and deception. To address this concern, this study combined behavioral assessments and neural measures using electroencephalography (EEG) to examine whether short-term perceptual training enhances people's ability to distinguish AI-generated from human speech. Thirty participants (of either sex) listened to sentences produced by human speakers and corresponding AI-generated clones, judging each sentence as either human or AI-generated before and after a brief (∼12 min) training session, during which voices were explicitly labeled as “human” or “AI.” Behaviorally, participants showed consistently poor discrimination before and after training, with only minimal improvement. However, neural analyses revealed substantial training-induced changes. Specifically, temporal response function (TRF) analysis identified significant neural differentiation between speech types at early (∼55 ms, ∼210 ms) and later (∼455 ms) auditory processing stages following training. Additional EEG analyses, including spectral power and decoding, were conducted to further investigate training effects, but these measures revealed limited differentiation. The findings here highlight a dissociation between behavioral and neural sensitivity: while listeners struggle to behaviorally discriminate sophisticated AI-generated voices, their auditory systems rapidly adapt to subtle acoustic differences following short-term exposure. Understanding this neural-behavioral dissociation is crucial for developing effective perceptual training protocols and informing policies to mitigate societal threats posed by increasingly realistic synthetic voices.

## Significance Statement

Artificial intelligence (AI)-generated voices are becoming increasingly indistinguishable from real human speech, raising serious concerns about fraud as scammers can convincingly impersonate trusted individuals. Our study shows that even when listeners cannot behaviorally distinguish AI-generated voices from real human voices, brief perceptual training enables their brains to detect subtle acoustic differences. Our findings thus reveal a dissociation between neural sensitivity and behavioral performance in recognizing AI-generated speech. By identifying this gap, we highlight an important opportunity: developing specialized training programs that guide listeners to recognize and utilize these subtle differences. Such targeted training could significantly enhance people's ability to identify synthetic voices, offering potential protection against the growing risks of scams and misinformation enabled by increasingly realistic AI speech technologies.

## Introduction

Significant advancements in artificial intelligence (AI)-based speech synthesis have led to the widespread adoption of synthetic voices in everyday applications (Ning et al., 2019), such as Apple's Siri and Microsoft's Cortana (Bentley et al., 2018; Ammari et al., 2019). These speech synthesis tools offer tangible benefits, for instance, by making text readily available to individuals with visual impairments or by providing convenient hands-free interaction. Yet with increasing accessibility comes growing concerns: the same technology that enables accessible communication may be exploited for malicious activities. For example, cybercriminals can use AI speech synthesis to pretend to be someone you know—such as colleagues, relatives, or friends—and commit fraud (Westerlund, 2019). Consequently, there is an urgent need to develop reliable methods and training procedures for human listeners to distinguish AI-cloned speech from genuine human voices (Mai et al., 2023).

Humans possess a remarkable ability to recognize individuals by their voices, an ability finely tuned to different listening contexts (McGehee, 1937; Latinus and Belin, 2011). Each person's voice contains unique acoustic features, such as pitch, accent, speaking rhythm (prosody), resonance, timbre, and articulation patterns, all of which listeners can use to identify who is speaking (Clifford, 1980; Belin, 2006; Belin et al., 2011; Bruder et al., 2024; Lavan et al., 2024). Voice recognition capabilities are partly innate—for example, newborn infants are able to distinguish unfamiliar voices and prefer their mother's voice (Spence and Freeman, 1996; Floccia et al., 2000)—and are partly learned in specific social and linguistic contexts (Goldstone, 1998; Eisner and McQueen, 2005). For example, listeners show improved voice recognition when hearing speakers in their native language because they can utilize subtle pronunciation differences among talkers (Perrachione and Wong, 2007; Perrachione et al., 2011). This suggests that, while our auditory system is inherently sensitive to acoustic differences between human voices, effectively using these differences for accurate speaker identification requires experience and training in specific contexts. Although extensive research continues on human voice recognition and related training (Eisner and McQueen, 2005; Samuel and Kraljic, 2009; Zhang et al., 2021), the rapid rise of highly realistic AI-generated speech introduces a new, unfamiliar AI speech context to our hearing brain.

Differentiating AI-generated speech from human speech presents a novel challenge for the human voice recognition system. Modern AI-based speech synthesis algorithms replicate critical voice features of human speech (Ning et al., 2019; Choi et al., 2020), enabling synthetic voices to frequently pass everyday “Turing tests,” where listeners often fail to identify them as artificial or “deepfake” (Müller et al., 2022; Mai et al., 2023; Groh et al., 2024; McGettigan et al., 2024; Barrington et al., 2025). Nevertheless, AI-generated speech fundamentally differs from human speech in its underlying production mechanisms: human voices emerge from physiological processes involving vocal-fold vibrations and dynamic vocal-tract modulations (Ghazanfar and Rendall, 2008; Kreiman and Van Lancker Sidtis, 2011; Zhang, 2016), whereas AI speech is algorithmically constructed from computational models and learned statistical patterns (Ning et al., 2019). Due to differences in speech production mechanics, we believe that acoustic differences between AI-generated and human speech undoubtedly exist, although they are not yet well documented to our knowledge. Consequently, listeners fail to detect these critical auditory differences—not because AI and human speech are indistinguishable at the sensory level—but likely because the human auditory system has not yet become tuned to recognize them at the behavioral level (Hochstein and Ahissar, 2002). This scenario parallels the phenomenon of “perceptual metamers,” where physically distinct stimuli (e.g., colors with differing spectral properties) appear identical due to insensitivity within our perceptual systems (Freeman and Simoncelli, 2011; McDermott et al., 2013; Feather et al., 2019). Similarly, AI-generated and human speech might appear identical—not from a lack of acoustic differences—but rather because our voice recognition system, specialized for human voices, presently overlooks these auditory distinctions.

Can the human voice recognition system, which is initially tuned for human voices, be retuned to reliably detect differences between human and AI-generated speech? Although AI-generated speech is relatively new, the problem of adapting our perceptual systems to novel distinctions is not new (Goldstone, 1998). Research on perceptual learning has consistently shown that human sensory systems can become sensitive to more subtle acoustic cues than those typically employed in everyday perceptual discrimination tasks (Goldstone, 1998; Hochstein and Ahissar, 2002; Samuel and Kraljic, 2009; Watanabe and Sasaki, 2015). For example, people can become experts at differentiating faces, birds, or other stimuli only after relevant training directs their attention to the subtle but key features (Diamond and Carey, 1986; Gauthier et al., 2000). In the field of speech perception, short-term perceptual learning has been shown to enhance perceptual sensitivity to nonnative languages and foreign-accented speech (Clarke and Garrett, 2004; Samuel and Kraljic, 2009). In the same vein, it is plausible that short-term exposure or perceptual training could help listeners reliably leverage acoustic differences, beyond those typically used for differentiating human voices, to distinguish between human and AI-generated speech.

Our study aims to investigate whether a brief period of perceptual training—an ecologically valid and practically feasible approach—can retune listeners’ auditory perception and enhance their ability to differentiate between AI-generated and human speech. We hypothesize that the auditory system inherently registers subtle acoustic differences between AI-generated and human speech, as reflected in distinct neural activities during auditory processing, given that detailed acoustic information is preserved within early auditory processing streams (Nahum et al., 2008; Ahissar et al., 2009). Nevertheless, listeners may initially fail to utilize this information for behavioral discrimination without specific perceptual training. Following perceptual training, we expect listeners’ sensitivity to AI–human speech differences to increase, resulting in improved behavioral discrimination. To test this hypothesis, we combine psychophysical measures of speech discrimination with electroencephalographic (EEG) recordings, capturing neural responses as listeners evaluate sentences produced by humans and AI-generated voices. Our findings will offer insights into whether—and how—short-term training modulates neural processing of AI-generated speech, informing targeted interventions and advancing our understanding of the neural mechanisms underlying human–machine voice discrimination.

## Materials and Methods

### Ethics statement

The study was approved by the Joint CUHK-NTEC Clinical Research Ethics Committee (CREC Ref. No. 2023.008). All participants provided written informed consent before completing any experimental interventions, and compensation was provided to each participant after the experiment was completed.

### Participants

Thirty-five healthy participants (age 20–32, 18 female participants) took part in the experiment. All participants were native Mandarin speakers, reported normal hearing, were not taking prescription drugs, and had no history of any neurological disorders or brain injuries. The final dataset included data from 30 participants (age 20–32, 15 females; all are right-handed). Five participants were excluded from the final dataset as there was a problem with the earphones during the experiment (*N* = 11, 14); we encountered technical issues during EEG recording for one participant (*N* = 17); and there were a considerable number of bad channels on the electroencephalogram (EEG) cap (*N* = 25, 31). None of the participants were familiar with or had previously interacted with the speakers whose voices were used in this study, ensuring no prior exposure to any of the speakers’ voices.

### Stimuli

#### Human speech stimuli generation

**Figure 1.**
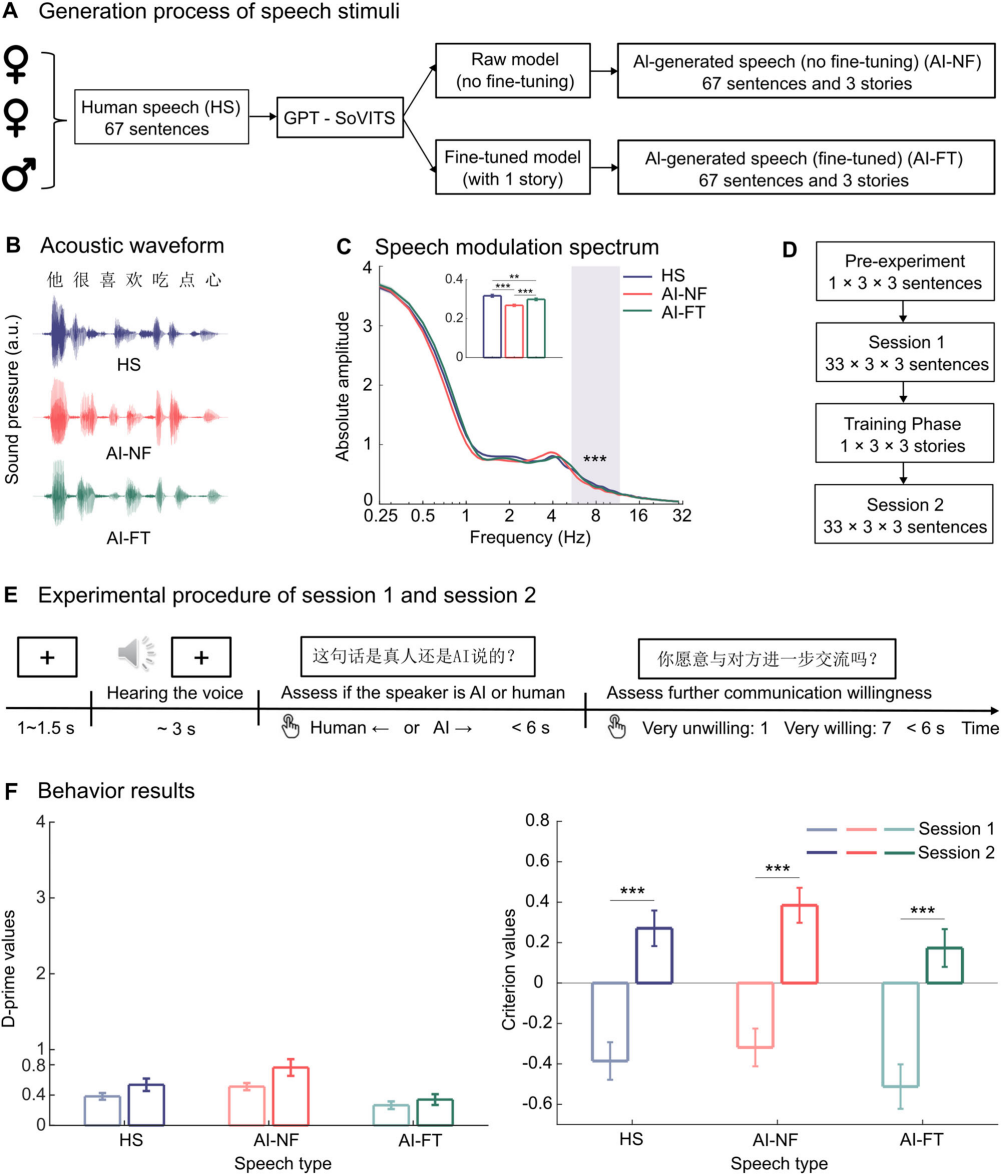
Stimulus generation, experimental paradigm, and behavioral results. ***A***, Generation process of speech stimuli. Three native Mandarin speakers (1 male, 2 female speakers) each recorded 67 sentences and one story summary, constituting the human speech dataset (HS). Using GPT-SoVITS, 67 sentences and 3 stories were directly synthesized without fine-tuning, categorized as AI-generated speech without fine-tuning (AI-NF). Following fine-tuning with one human-produced speech sample, the model generated 67 sentences and 3 stories, categorized as fine-tuned AI-generated speech (AI-FT). The voices of the three speakers were generated independently for each condition. ***B***, Acoustic waveform of the utterance. The acoustic waveform corresponds to the Mandarin utterance “他很喜欢吃点心” (He enjoys eating desserts) produced by the male speaker. The *y*-axis represents the amplitude of the sound pressure level in arbitrary units (a.u.). ***C***, Speech modulation spectrum of various types of speech signals. The shaded box indicates the cluster that exhibited significant effects following a cluster-based permutation test (**p < 0.01; ***p < 0.001). The inset bar graphs illustrate the average modulation spectra of the identified cluster within the frequency band of 5.4–11.7 Hz. ***D***, Experimental paradigm of the entire experiment. ***E***, Trial paradigm for presenting speech stimuli during EEG recording. ***F***, Plots of the behavioral data. The left panel shows D-prime values that were calculated to quantify the performance of speech type detection. The right panel shows criterion values that were calculated to represent the judgmental tendencies of the subjects. Error bars indicate the mean and standard error of the mean, black stars indicate a significant difference (repeated-measures ANOVA, FDR-adjusted) between two sessions.

To train the AI speech synthesizer and provide perceptual training materials, we selected four literary Chinese adaptations of internationally recognized fairy tales: *Little Red Riding Hood*, *The Little Match Girl*, *Cinderella*, and *The Wild Swans*. Each story was manually summarized into a standardized version of ∼350 words, with the essential narrative components carefully retained to maintain the original meaning and structure. For testing participants’ ability to differentiate human from AI-generated speech, we further selected 67 standardized Mandarin sentences from the Mandarin Speech Perception Test (MSP; Fu et al., 2011). The distribution of vowels, consonants, and tones within these sentences closely reflected those in commonly spoken Mandarin. Example sentences are illustrated in Figure 1*B*. The selected materials were recorded in a soundproof room by two female and one male speaker, all speaking standard Mandarin at a natural conversational pace. Each speaker recorded all 67 sentences and one story summary (*Little Red Riding Hood*). Recordings were digitally captured in .wav format at a sampling rate of 44.1 kHz and verified for naturalness.

#### AI speech stimuli generation

AI-generated speech was synthesized using GPT-SoVITS (see https://github.com/RVC-Boss/GPT-SoVITS). GPT-SoVITS is an open-source, few-shot voice conversion and text-to-speech (TTS) tool. It enables the synthesis of highly realistic AI-generated voices (validated in our behavioral findings; Fig. 1*F*) with minimal or no fine-tuning data and requires little computational power. In this study, the synthesizer (GPT-SoVITS-V3 model, which is state-of-the-art at the time) was fine-tuned, or not, using the human recordings of *Little Red Riding Hood*, subsequently producing two types of AI-generated voices: fine-tuned AI speech (AI-FT) and non-fine-tuned AI speech (AI-NF). The fine-tuning process followed the model's default settings, allowing for a direct comparison between the two speech variants. The fine-tuned version can better imitate the target human speaker, while the non-fine-tuned version, though it still sounds human and bears a resemblance to the target speaker, has lower quality than the fine-tuned version.

These synthesized voices were used to generate the 67 Mandarin sentences and the other three story summaries, as shown in Figure 1*A*. The fine-tuned AI speech (AI-FT) was generated after extensive training with the *Little Red Riding Hood* recordings, while the non-fine-tuned AI speech (AI-NF) was generated without additional fine-tuning, relying solely on short samples (sentences or summaries).

All speech stimuli (human and AI-generated) were digitally captured in .wav format, downsampled from 44.1 to 16 kHz, and amplitude-normalized to 70 dB SPL using a reference pink noise recorded and calibrated in the EEG testing environment.

#### Acoustic analysis of stimuli

The speech modulation spectrum is a prominent feature in speech perceptual analysis, which is widely used to characterize the temporal structure of speech at different modulation rates (Giraud and Poeppel, 2012). Specifically, modulation spectra have been shown to capture the rhythmic properties of speech that are crucial for speech perception and neural entrainment. Evidence shows that the human auditory system is specialized for the long-term modulation spectrum and employs neural mechanisms to extract essential acoustic features from speech (Teng et al., 2021). To quantify acoustic characteristics, facilitate subsequent analyses, and compare acoustic differences among fine-tuned AI-generated, non-fine-tuned AI-generated, and human-produced speech stimuli, we employed a straightforward approach: extracting the speech amplitude envelopes and computing their modulation spectra. If this basic method can already reveal salient acoustic differences among the speech types, it would support our hypothesis outlined in the introduction—that acoustic differences indeed exist between AI-generated and human speech, providing potential auditory cues for listeners to learn to differentiate between these speech types. Admittedly, the observed differences here reflect only the specific stimulus materials used in our study, but they nevertheless offer preliminary evidence supporting our hypothesis.

First, speech stimuli were filtered through a gammatone filterbank consisting of 32 logarithmically spaced frequency bands, covering a range from 50 to 8,000 Hz (Patterson et al., 1988; Ellis, 2009). Subsequently, the envelope of each frequency subband was obtained by applying the Hilbert transform on each band and computing the absolute values (Glasberg and Moore, 1990; Søndergaard and Majdak, 2013). The amplitude envelope of each frequency band was extracted and downsampled to 100 Hz, aligning with the subsequent EEG data sampling rate and thus aiding subsequent analyses. The amplitude envelope was then averaged across the 32 frequency subbands for each speech sentence and speaker in each speech type. Finally, we applied fast Fourier transform (FFT) to these averaged envelopes to derive modulation spectra for each speech type. The modulation spectra were computed by averaging across all 198 speech stimuli (33 sentences × 3 speakers × 2 sessions) for each speech type. The analyses were conducted using MATLAB R2022a.

To examine differences in acoustic features across three speech types, we conducted a cluster-based permutation test on the modulation spectra (0–30 Hz) of 198 speech stimuli (33 sentences × 3 speakers × 2 sessions). The analysis was performed using the ft_freqstatistics function from the FieldTrip toolbox in MATLAB. Specifically, we used the one-tailed independent samples *F* statistic (ft_statfun_indepsamplesF) to evaluate differences between conditions. Clusters are defined under the threshold of 0.05 for spatial dimensions by using the Monte Carlo Method to calculate the significance probability. The test statistic used in the permutation distribution was the maximum cluster sum (maxsum), and the number of random permutations was set to 5,000 to ensure robust estimation of the null distribution. Indeed, as we suspected, the modulation spectra showed differences between human-produced speech, fine-tuned AI-generated speech, and non-fine-tuned AI-generated speech (Fig. 1*C*).

### Experimental procedure and EEG recording

#### EEG recording procedure

EEG recordings were conducted in a soundproof experimental chamber with dim lighting in the communal lab of the Department of Psychology. Participants sat comfortably throughout the experiment. EEG signals were captured using a 64-electrode elastic cap (ANT Neuro, waveguard original) equipped with Ag/AgCl electrodes, comprising 61 scalp electrodes, a nose-tip reference electrode (originally M1), and a ground electrode positioned between Fpz and Fz. Signals were filtered with a 0.1–100 Hz online bandpass filter and recorded at a sampling rate of 500 Hz, with electrode impedances maintained below 20 kΩ. Auditory stimuli were binaurally delivered through EEG-compatible insert earphones (ER-3C) at ∼70 dB SPL. Stimulus presentation and behavioral responses were managed using MATLAB-based Psychtoolbox-3 (Brainard, 1997; Kleiner et al., 2007).

#### Experimental procedure

The experiment consisted of one pretest session, two main experimental sessions, and an intervening training session (Fig. 1*D*).

During EEG setup, participants underwent a brief pretest session designed to familiarize them with the task and auditory environment, involving nine sentences (1 sentence × 3 speaker conditions × 3 speech conditions) not used in subsequent sessions. Participants were instructed to remain relaxed, minimize blinking, limit head and body movement, and maintain visual focus at the screen's center. Behavioral and EEG data from the pretest were not recorded.

In the first main experimental session, participants listened to 297 randomly ordered sentences (33 sentences × 3 speaker conditions × 3 speech conditions) without knowing the speaker's identity (human or AI-generated). Each trial began with a yellow fixation cross displayed for 1–1.5 s, followed by the auditory sentence stimulus. Upon sentence completion, participants responded to two questions: (1) identifying the speaker as human or AI (Was the sentence spoken by a real person or an AI? Left arrow for human, right arrow for AI) and (2) rating willingness to further communicate (Are you willing to communicate with them further?) on a 7-point Likert scale (1 = Very unwilling; 7 = Very willing). We did not further analyze the rating responses as it was not related to our central question. This session lasted ∼25 min (Fig. 1*E*).

Following a brief rest of several minutes, the training session began, introducing participants to 9 narrative recordings (3 speaker conditions × 3 speech conditions, ∼90 s each), explicitly labeled as human or AI-generated to facilitate perceptual learning of distinguishing features. Each recording was followed by a 1–1.5 s silent interval, automatically progressing without requiring behavioral responses. The training phase lasted ∼12 min.

The second experimental session mirrored the first but utilized a separate set of 33 novel sentences, not used in the first session, to prevent familiarity effects.

EEG and behavioral responses were recorded throughout the experimental and training sessions, with participants permitted brief breaks between sessions. Overall, the duration of the experiment was ∼60 min.

### Data analysis

We first provided an overview of our analyses here. The present study aimed to investigate whether listeners can distinguish between human-produced and AI-generated speech at both behavioral and neural levels and to determine if short-term perceptual training enhances such differentiation. To comprehensively explore neural encoding of speech differences, we conducted behavioral analyses, as well as neural analyses using three distinct methods—temporal response function (TRF) analysis, EEG spectral analysis, and EEG decoding—to quantify different aspects of neural processing and examine how speech distinctions might be encoded in neural signals from multiple perspectives. Specifically, we hypothesized that (1) at the behavioral level, participants would demonstrate limited ability to distinguish between human-produced and AI-generated speech, but short-term training might enhance their performance; (2) at the neural level, TRFs would reveal temporal neural tracking differences posttraining (Teng et al., 2017, 2019, 2024); (3) EEG spectral analyses would identify global neural differences between speech types across entire sentence stimuli (Teng et al., 2017, 2020); and (4) EEG decoding analyses would reveal whether spatial EEG patterns provide discriminative spatial neural codes differentiating human from AI-generated speech (Cichy et al., 2014).

#### Behavioral data analysis

Behavioral data were analyzed within the framework of signal detection theory (Macmillan and Creelman, 1991) in MATLAB R2022a (The MathWorks; RRID: SCR_001622) using the Palamedes toolbox 1.5.1 (RRID: SCR_006521; Prins and Kingdom, 2018). Statistical analyses were also conducted using IBM SPSS Statistics 28.0.1.0 (RRID: SCR_016479). Speech can be perceived by category, and signal detection theory is widely applied in perceptual detection paradigms to quantify the perceptual sensitivity and decision bias (Gerrits and Schouten, 2004; Teng et al., 2017). In the experimental sessions, for each speech condition, a two-by-two confusion matrix was constructed by treating the trials of a specific speech type as “target” and the trials of other types as “noise.” Specifically, when the real speech stimulus was treated as “target,” the other two types of AI speech stimuli (i.e., fine-tuned and non-fine-tuned) were pooled together and regarded as “noise.” Alternatively, when one type of AI speech stimulus (e.g., fine-tuned) was treated as the “target,” the other type of AI speech stimulus is discarded and the real speech stimulus was defined as “noise.” In the current analysis, the correct detection of the speech type in the target trials was counted as a “hit,” while the correct detection of the speech type in the noise trials was recorded as “correct rejection.” The *d*’ values and criterion values for each speech condition were computed using the hit rates and false alarm rates from their respective confusion matrices under the following formula (Macmillan and Creelman, 1991):

$$d^{\prime }\;=\;z(H)\;-\;z(F),$$

c=−0.5(z(H)+z(F)).

#### EEG preprocessing

EEG data analysis was conducted in MATLAB R2022a using the FieldTrip toolbox 20230118 (RRID: SCR_004849; Oostenveld et al., 2011), the wavelet toolbox and the multivariate temporal response function (mTRF) toolbox (Crosse et al., 2016).

EEG recordings were offline referenced to the average activity across all 61 electrodes. Raw EEG data were further processed using a bidirectional (two-pass) fourth-order Butterworth infinite impulse response (IIR) filter, applying a 1 Hz high-pass and a 45 Hz low-pass cutoff frequency. This configuration followed the default setting of the FieldTrip toolbox. After filtering, the data were downsampled to 100 Hz to reduce computational load. Trials were visually inspected to identify and exclude artifacts, such as channel jumps or substantial signal fluctuations. Independent component analysis (ICA; Hyvärinen and Oja, 2000) was then applied to remove artifacts resulting from eyeblinks, eye movements, and cardiac activity. Following preprocessing, no more than 30 trials per experimental session were excluded, retaining over 90% of trials for further analysis. Data from the training session were fully retained. In experimental sessions, each trial was segmented into a 6.5 s epoch, encompassing a 1.5 s prestimulus interval and a 5 s poststimulus interval (the longest sentence stimuli lasting <3 s). Baseline correction was performed by subtracting the mean signal amplitude from the −1 to 0 s interval preceding stimulus onset. To examine neural tracking across specific frequency bands, EEG waveforms were further filtered using a Butterworth IIR bandpass filter (order of 4, applied forward and backward to compensate for filter delay) for the delta (1–3 Hz), theta (4–7 Hz), and alpha (8–13 Hz) bands. We did not analyze the EEG data from the training session.

#### EEG spectral analysis

The rationale for conducting EEG spectral analysis is grounded in the hypothesis that neural oscillations rhythmically track the temporal structure of speech (Luo and Poeppel, 2007; Poeppel and Assaneo, 2020). As illustrated in Figure 1*C*, the modulation spectra of the speech stimuli display differences between AI and human speech. If the auditory brain follows this rhythmic tracking mechanism, neural responses should reflect corresponding spectral peaks in the EEG signal (Teng et al., 2020), potentially encoding differences between speech stimulus types (AI-generated vs human-produced). To test this hypothesis, we performed amplitude spectral analysis on EEG responses.

Epochs were precisely trimmed to retain EEG signals corresponding solely to periods containing actual speech sounds, excluding any preceding silence. This ensured that the EEG segments were temporally aligned with the exact duration of their corresponding speech stimuli and reduced the influence of the onset neural response. To minimize spectral leakage and reduce the impact of speech onset, we applied a Hanning window to each EEG segment after removing the initial 100 ms of data. Subsequently, the EEG data were transformed into the frequency domain via FFT, zero-padded to 400 samples to enhance frequency resolution. To examine potential neural differences across the spectral range relevant to speech envelope tracking, we analyzed EEG power across a broad 1–15 Hz range. Single-trial spectra were averaged within each stimulus type and experimental session to produce the mean amplitude spectrum for each speech type.

#### Temporal response functions analysis

The analyses in the spectral domain, such as amplitude spectral analysis, primarily quantify the distribution of signal power across different frequency components but do not fully capture the temporal details of how neural responses track acoustic variations. To investigate whether the auditory system neurally distinguishes speech types and to examine the temporal dynamics of neural tracking, we computed TRFs. TRFs have been widely used in neurophysiological studies on speech perception to characterize how neural responses track and encode the acoustic envelopes of speech stimuli temporally (Di Liberto et al., 2015; Wang et al., 2019; Teng et al., 2021; Lindboom et al., 2023).

TRFs model the relationship between neural responses and the amplitude envelopes of speech stimuli, averaged across cochlear bands (see above, Acoustic analysis of stimuli, for details), through ridge regression implemented in the mTRF toolbox (Lalor et al., 2006; Ding and Simon, 2012; Crosse et al., 2016). The TRF reconstruction establishes a linear mapping between the neural response and the original speech stimulus. This relationship can be estimated using ridge regression with a parameter *λ* to control for overfitting. The estimation of the TRF is performed in the following matrix format:

$$\mathrm{TRF}=(S^{T}S+\lambda I{)}^{-1}S^{T}r.$$

The variable *S* is the lagged time series of the amplitude envelopes of the sentence stimuli. The time lags extend from −300 ms (prestimulus baseline) to +600 ms (poststimulus response) relative to auditory onset markers. The regularization parameter for all trials per subject was empirically chosen as 100, which is the lowest value such that any increase would result in no visible improvement in the plotted estimate (Lalor et al., 2006). The variable *r* is the temporally aligned EEG data, ranging from −300 to +600 ms relative to auditory stimulus onset, capturing neural responses to sentence stimuli. The stimulus and response signals were uniformly resampled to a common sampling rate of 100 Hz. The amplitude envelopes are normalized to have a variance of 1 to ensure consistency in their scale while preserving their overall shape, and the EEG signals are channel-wise *z*-score normalized to have a mean of 0 and a variance of 1 to achieve scale alignment across participants. TRF weights were estimated via individual trial-based training. Then, we computed standardized averages of TRF weights across all sentences within each speech condition.

**Figure 2.**
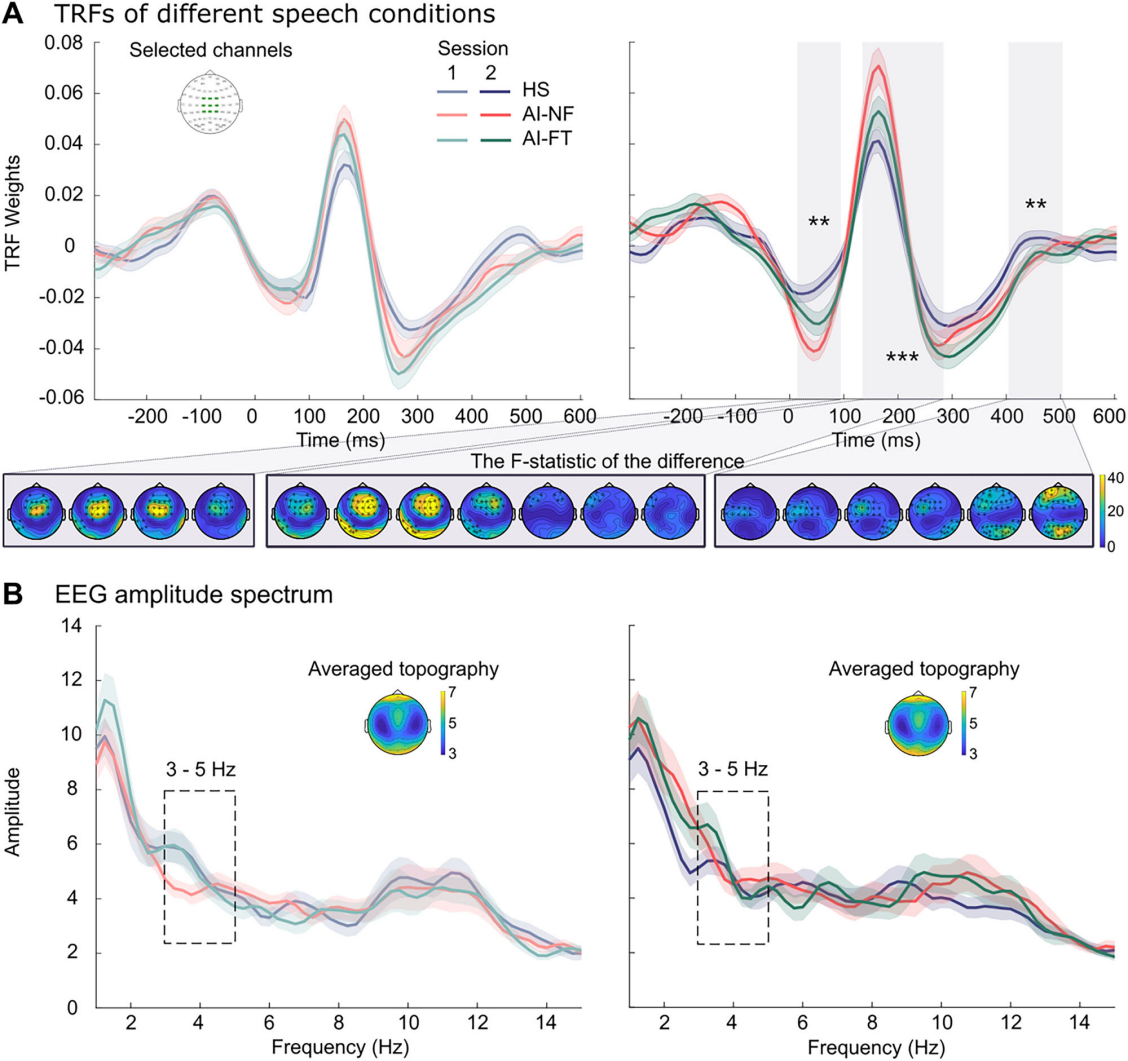
Neural tracking of different speech signals. ***A***, Temporal response function (TRF) result averaged over the selected channels (FCz, FC1, FC2, Cz, C1, C2, CPz, CP1, CP2—the green regions on the inserted topographies). The left and right panels represent the results of Session 1 and Session 2, respectively. We identified three periods (shaded boxes) showing significant differences across all speech conditions in Session 2. Black stars indicate a significant difference (**p < 0.01; ***p < 0.001). The bottom panel illustrates the largest channel-time clusters that exhibit statistically significant differences across the three speech conditions, plotted on top of the *F* statistic of the difference. Black stars represent the electrodes that show significant differences in the contrasts. ***B***, Frequency spectrum of the neural signal averaged over the selected channels (Cz and FCz). The left and right panels represent the results of Session 1 and Session 2, respectively. Topological maps represent the averaged amplitudes of the three speech conditions within the frequency range of 3–5 Hz.

For subsequent analysis and visualization, we focused on a predefined cluster of electrodes in the central scalp region (FCz, FC1, FC2, Cz, C1, C2, CPz, CP1, CP2; Fig. 2*A*). This specific region was selected based on its high sensitivity to auditory-evoked neural activity. The central scalp region has been associated in previous studies with a distributed network engaged in speech processing, including pathways often described as anteroventral and posterodorsal streams, as well as higher-level integration processes (Hickok and Poeppel, 2007; Rauschecker and Scott, 2009). Furthermore, these electrodes were selected as they typically exhibit a relatively high signal-to-noise ratio (SNR) in EEG recordings, which is crucial for obtaining reliable and interpretable TRFs.

Ultimately, this neural encoding framework allowed us to identify temporal patterns of neural activity associated with different speech types and to investigate how the auditory system encodes dynamic acoustic features of human and AI-generated speech.

#### Cluster-based permutation test

To compare neural responses across speech conditions, we conducted cluster-based permutation tests (Maris and Oostenveld, 2007) with a significance level of 0.05 individually for temporal (TRFs 0–500 ms) and spectral (1–15 Hz frequency spectrum) features. The null hypothesis for this cluster-based permutation test is that the three conditions (HS, AI-NF, and AI-FT) do not show any statistically significant differences in individual measures.

For all analyses, surrogate datasets were generated by permuting condition labels within subjects across 5,000 iterations, preserving within-subject dependencies while randomizing condition assignments. This approach controls for intersubject variability, and in each permutation, the point-to-point one-tailed *F* statistic was computed (using ft_statfun_depsamplesFmultivariate in FieldTrip).

Clusters are defined under the threshold of 0.05 for spatial dimensions by triangulating the electrode positions of the EEG montage (with a minimum of 3 adjacent channels required for inclusion). The actual test statistic is defined as the maximum of the cluster-level statistics. Each cluster-level statistic is calculated as the sum of the sample-specific *F* statistic within a given cluster. The largest of these cluster-level statistics across all clusters is then used as the actual test statistic.

#### Decoding analysis

In the decoding analysis, we utilized the high-performance LIBLINEAR library (Fan et al., 2008) implemented in MATLAB, specifically using the L2-regularized L2-loss (dual) support vector machine (SVM) classifier. EEG data were segmented into epochs of 1.5 s, beginning 1 s before sentence offset and extending 500 ms afterward. Because each sentence varied in length, we aligned the EEG epochs to the sentence endings, assuming participants would have accumulated sufficient acoustic information by that time to determine whether the voice was human or AI generated. Aligning epochs to sentence offsets therefore optimizes decoding efficiency, since minimal distinguishing information is typically available at sentence onset.

Prior to decoding, EEG data for each participant were *z*-score normalized, resulting in a mean of 0 and a standard deviation of 1 for each channel across all selected time points to control for individual variability. To ensure robust performance evaluation and unbiased hyperparameter selection, we employed a nested 10-fold cross-validation strategy for each participant. The dataset was first randomly permuted and partitioned into 10-folds of equal size. In the outer loop, each fold was iteratively held out as the test set, while the remaining ninefolds were used for training. In the inner loop, for each training set, an additional 10-fold cross-validation was performed to optimize the SVM hyperparameter *c* by searching from 2^−5^ to 2^15^ with a step size of 2 in powers of 2. The above nested 10-fold cross-validation was performed independently for each time point to evaluate how decoding accuracy evolved temporally. Decoding accuracy (percent correct classification) was computed at each time point and averaged across trials within each condition, allowing for comparisons between experimental conditions. To prevent trial selection biases, the final decoding accuracy was reported as the average classification accuracy across all outer-loop test folds.

## Results

The current study investigates whether listeners can distinguish authentic human speech from AI-generated speech at both behavioral and neural levels and whether brief perceptual training enhances their ability to do so. At the behavioral level, we quantified listeners’ sensitivity (*d*’) and response biases to determine how well they differentiate human voices from AI clones before and after training. At the neural level, we analyzed EEG recordings using TRFs to capture neural tracking of dynamic acoustic differences between speech conditions over time. Additionally, we conducted frequency–domain analyses to examine neural differences across speech stimuli at the scale of entire sentences. Finally, we performed EEG-based decoding analyses, leveraging spatial patterns across electrodes, to uncover possible spatially distributed neural representations distinguishing AI-generated from human speech.

### Behavioral discrimination of human and AI speech

We first examine how well listeners can distinguish human speech from AI-generated speech. In the behavioral test, participants were asked to categorize human speech and AI-generated speech by button press. The behavioral sensitivity in distinguishing these two speech types provides a first indication of whether listeners exhibit a distinct preference for human speech over the AI-generated speech.

We first examine listeners’ discrimination sensitivity (*d*’) in distinguishing different speech types. Participants exhibited consistently low sensitivity in distinguishing between human and AI-generated speech (Fig. 1*F*), as indicated by low discrimination sensitivity in both pretraining (Session 1) and posttraining (Session 2) experimental sessions (Session 1: human speech, *M* = 0.383, SE = 0.044; non-fine-tuned AI speech, *M* = 0.512, SE = 0.047; fine-tuned AI speech, *M* = 0.266, SE = 0.050. Session 2: human speech, *M* = 0.536, SE = 0.082; non-fine-tuned AI speech, *M* = 0.764, SE = 0.110; fine-tuned AI speech, *M* = 0.341, SE = 0.071). A Speech type × Session repeated-measures ANOVA revealed main effects of Speech type on *d*’ (*F*_(2,58)_ = 65.725, *p* < 0.001, *η_p_*^2^ = 0.694). In contrast, the main effect of Session on *d*’ was not significant (*F*_(1,29)_ = 3.918, *p* = 0.057, *η_p_*^2^ = 0.119), indicating that the brief training only yielded minimal improvement in behavioral performance. Furthermore, a significant interaction between Speech type and Session was observed (*F*_(2,58)_ = 5.314, *p* = 0.008, *η_p_*^2^ = 0.155). To explore this interaction, paired *t* tests were conducted as post hoc analyses to examine the simple effects of Session within each speech condition. The results revealed no significant differences in *d*’ between the two sessions for any of the speech types after applying false discovery rate (FDR) correction (human speech: *t*_(29)_ = −1.975, *p* = 0.087, *d* = −0.361; non-fine-tuned AI speech: *t*_(29)_ = −2.380, *p* = 0.072, *d* = −0.435; fine-tuned AI speech: *t*_(29)_ = −1.022, *p* = 0.315, *d* = −0.187, paired *t* test, FDR*-*corrected). Notably, prior to applying the FDR correction, a statistically significant training effect was observed in the discrimination of non-fine-tuned AI speech (*p* = 0.024).

Further analysis of the criterion, which measures participants’ decision bias, revealed a marked transition from negative values in Session 1 to positive values in Session 2 (Fig. 1*F*). A Speech type × Session 2-way rmANOVA revealed main effects of Speech type (*F*_(2,58)_ = 31.937, *p* < 0.001, *η_p_*^2^ = 0.524) and Session (*F*_(1,29)_ = 33.108, *p* < 0.001, *η_p_*^2^ = 0.533) on criterion. However, the main effect of the interaction between Speech type and Session was not significant (*F*_(2,58)_ = 0.302, *p* = 0.607, *η_p_*^2^ = 0.010). Planned comparisons using paired *t* tests with adjusted FDR correction (Benjamini and Hochberg, 1995) on the main effect of Session showed that the criterion in Session 2 is significantly larger than that in Session 1 among all the different speech types (human speech: *t*_(29)_ = −5.653, *p* < 0.001, *d* = −1.032; non-fine-tuned AI speech: *t*_(29)_ = −5.905, *p* < 0.001, *d* = −1.078; fine-tuned AI speech: *t*_(29)_ = −5.089, *p* < 0.001, *d* = −0.929, paired *t* test, FDR corrected). This result revealed a shift from a tendency to identify presented stimuli as signals more frequently in Session 1 to a conservative bias (a tendency to identify as non-signals more frequently) in Session 2 when treating each type of speech as signal.

Our findings indicate that while perceptual training did not significantly enhance participants’ behavioral sensitivity, it did influence their response strategies. One potential explanation for this shift in response criterion is that participants learned during training that most speech samples (two-thirds) were AI generated, making them more likely to categorize sentences as AI speech in the second session. Alternatively, participants may have become familiar with subtle acoustic differences between human and AI speech during training, leading them to adopt a more conservative criterion for identifying AI speech rather than assuming speech samples were human. If the observed criterion change resulted purely from participants’ awareness of stimulus proportions, we would not expect subsequent neural analyses to reveal speech-related neural differences. However, if participants indeed learned to perceive acoustic distinctions during training, neural differences between speech types should emerge in subsequent analyses.

### Acoustic differences of speech types in modulation spectra

Temporal envelopes derived from broad frequency bands of the speech signal are known to carry the information critical for speech recognition (Shannon et al., 1995). Since neural responses to temporal modulations have been found functionally relevant to the processing of speech and music (Di Liberto et al., 2015; Doelling and Poeppel, 2015), we employed modulation spectrum analysis and conducted a cluster-based permutation test to uncover subtle acoustic differences that might influence perception. Averaged modulation spectra across all stimuli in both sessions revealed a consistent spectral peak ∼4 Hz among the three speech conditions (Fig. 1*C*). We further applied a cluster-based permutation approach to the full spectral range to capture potential differences in power across a broader temporal and spectral context. This analysis revealed a statistically significant cluster in the frequency range of 5.4–11.7 Hz (cluster *F* statistic = 351.47, *p* < 0.001), indicating a significant difference in modulation characteristics between AI-generated and human speech within this band.

In the post hoc analysis, the modulation spectra within the frequency ranges of the identified significant clusters were averaged for each sentence stimulus. Subsequently, paired *t* tests with FDR correction were conducted to examine pairwise differences among speech conditions. The results revealed that both human speech (*t*_(197)_ = 7.359, *p* < 0.001, *d* = 0.523) and fine-tuned AI speech (*t*_(197)_ = −5.666, *p* < 0.001, *d* = −0.403) exhibited significantly higher modulation spectra compared with non-fine-tuned AI speech. Moreover, a significant difference was also observed between human speech and fine-tuned AI speech, with human speech showing a higher modulation spectrum (*t*_(197)_ = −2.985, *p* = 0.003, *d* = −0.212). Indeed, acoustic differences between AI-generated and human speech are clear, but the crucial question is whether the auditory system actually tunes into these cues to discriminate between them.

### Neural differentiation of speech types through TRF analysis

Although behavioral responses showed no significant differentiation between human and AI-generated speech, neural differences may still exist. To address this, we conducted TRF analyses to examine neural tracking before and after training. Cluster-based permutation tests applied to the averaged TRFs over participants (*n* = 30) from pretraining and posttraining revealed three prominent response components at ∼50, 200, and 300 ms after stimulus onset.

Cluster-based permutation tests comparing TRFs across the three speech conditions revealed no significant differences before training (Session 1). However, posttraining analyses (Session 2) identified three significant spatiotemporal clusters differentiating speech types, centered ∼55 ms (cluster *F* statistic = 3,245.07, *p* = 0.008), 210 ms (cluster *F* statistic = 5,732.34, *p* < 0.001), and 455 ms (cluster *F* statistic = 4,529.82, *p* = 0.002). These results indicate that short-term training induced neural differentiation among speech types, particularly evident in early (∼55 ms, ∼210 ms) and later (∼455 ms) auditory processing stages, despite participants’ inability to behaviorally differentiate between speech types. This suggests that the auditory brain can rapidly adapt to subtle acoustic distinctions between human and AI-generated speech, even though these neural adaptations do not immediately translate into improved behavioral discrimination abilities.

### No significant spectral differences between human and AI-generated speech

The TRFs investigate neural responses to acoustic dynamics over brief, local temporal scales; however, it is also informative to examine how neural responses differ globally across entire sentence stimuli, in the spectral domain. Better neural differentiation may emerge at the sentence scale. To characterize the neural oscillatory dynamics associated with different speech types, we first conducted a broad-spectrum analysis of EEG power across the 1–15 Hz range. Results from the cluster-based permutation tests indicated no significant differences among speech conditions in neither Session 1 (two positive clusters were identified: cluster *F* statistic = 142.82, *p* = 0.459; cluster *F* statistic = 48.9, *p* = 0.846) nor Session 2 (three positive clusters were identified: cluster *F* statistic = 321.5, *p* = 0.113; cluster *F* statistic = 129.77, *p* = 0.494; cluster *F* statistic = 44.67, *p* = 0.849) in terms of EEG spectral analyses.

Motivated by our acoustic modulation spectrum analysis, which identified a statistically significant acoustic cluster between speech types specifically within the 5.4–11.7 Hz band, we conducted a targeted follow-up analysis to evaluate whether these acoustic differences were manifested in neural power. Even within this acoustically driven frequency range, no significant modulations of EEG power were observed between conditions in either session (a positive cluster was identified in Session 1: cluster *F* statistic = 48.9, *p* = 0.589; no positive cluster was identified in Session 2). These findings suggest that the acoustic differences observed in the modulation spectra did not elicit corresponding changes in the magnitude of low-frequency neural oscillations.

### Limited EEG spatial differentiation of human and AI speech

In the TRFs analysis, we revealed distinct posttraining temporal neural tracking patterns, indicating differences in how neural activity follows the acoustic features of human versus AI-generated speech over time. However, previous EEG analyses were conducted on individual EEG electrodes in the temporal and spectral domains. It is possible that AI–human speech differences could be coded across EEG electrodes (e.g., EEG topographies), reflecting spatial neural codes of AI–human speech information (King et al., 2016). To examine this possibility, we conducted a decoding analysis, classifying speech types at each time point using data from all 61 electrodes and leveraging the topographical information across electrodes. Additionally, we filtered EEG signals into three frequency bands—delta, theta, and alpha—and applied the decoding algorithm (see Materials and Methods section), as these bands have been shown to encode different speech information (Giraud and Poeppel, 2012; Teng and Poeppel, 2019). The subject-averaged decoding accuracy fluctuated around chance level (∼50%). Paired *t* tests conducted at each time point from sentence onset to offset revealed no significant differences after correcting for multiple comparisons. This suggests that neural signals recorded by EEG lack sufficient discriminative topographical information across electrodes to differentiate neural responses to AI-generated versus real speech stimuli.

## Discussion

The present study investigated whether listeners can distinguish human speech from AI-generated speech behaviorally and neurally and examined the impact of short-term perceptual training on this ability. Behaviorally, participants consistently showed poor sensitivity (low *d*’) in distinguishing between human and AI-generated speech (Fig. 1*F*, left panel). Although the brief perceptual training did not significantly improve behavioral discrimination, it did influence response strategies, shifting participants toward a more conservative criterion—making them more likely to classify sentences as AI-generated after training (Fig. 1*F*, right panel). Neurally, however, significant training-induced differences emerged: TRF analyses revealed clear posttraining distinctions between speech types at early (∼55 ms, ∼210 ms) and late (∼455 ms) auditory processing stages (Fig. 2*A*). In contrast, complementary analyses of EEG spectral power and spatial decoding yielded no significant differences between speech conditions (Fig. 2*B*). Together, these results highlight a notable dissociation between neural sensitivity and behavioral performance in distinguishing human from AI-generated speech.

In the analysis of acoustic differences among speech types, we identified a statistically significant cluster spanning the modulation spectrum between 5.4 and 11.7 Hz. The frequency range of 5.4–11.7 Hz captures the mid-to-high modulations in speech, representing the rapid phonemic transitions and syllabic onsets that facilitate neural tracking of the speech units (Ding et al., 2017; Varnet et al., 2017; Poeppel and Assaneo, 2020). These modulations reflect the nonlinear and dynamic temporal structure of natural speech, including rapid spectral changes and natural pitch contours, which are critical for the naturalness of speech. Regarding AI-generated speech, although it is high fidelity it may fail to capture such rapid, dynamic fluctuations inherent in natural human speech (Wu et al., 2012; Paul et al., 2017; Müller et al., 2022). This absence may account for the significant differences observed in the 5.4–11.7 Hz modulation spectrum between AI-generated and human speech. Cortical oscillations are known to be highly sensitive to the multi-timescale, quasi-rhythmic structure of speech, enabling them to dynamically track speech-related neural activity (Giraud and Poeppel, 2012). Previous research indicates that speech perception operates on distinct timescales, with neural oscillations in the theta band (∼4–8 Hz) tracking syllabic rhythms (Poeppel, 2003; Luo and Poeppel, 2007), and in the alpha-beta range (8–30 Hz) tracking phonemic information (Giroud et al., 2024). These oscillations are known to synchronize with the temporal structure of speech, facilitating the parsing of linguistic units at different levels. The differences in the modulation spectrum between 5.4 and 11.7 Hz may lead to cortical phase resetting, excitatory alignment, and alterations in hierarchical information transmission (Giraud and Poeppel, 2012). These changes could, in turn, influence the neural coupling patterns and provide a potential mechanism for detecting differences between AI and human speech.

The observed dissociation between behavioral discrimination and neural differentiation highlights that the auditory system can rapidly adapt following brief perceptual training to detect subtle acoustic differences between human and AI-generated speech, even though these differences do not significantly affect behavioral judgments. This aligns with previous studies and theoretical frameworks such as reverse hierarchy theory (Nahum et al., 2008; Ahissar et al., 2009), which suggests that early sensory systems inherently capture detailed information, but focused training is necessary to utilize such sensory details for behavioral decisions. Initially, listeners did not discriminate well between human and AI-generated speech because their auditory systems are naturally tuned to differences among human voices rather than between human and synthetic speech. However, after ∼12 min of perceptual training, neural analyses (TRFs) clearly demonstrated enhanced neural sensitivity to these distinctions. These findings provide an optimistic outlook on realistic AI-generated (“deepfake”) speech concerns, suggesting humans inherently possess the neural capability to distinguish AI-generated from authentic human speech but require brief training to tune their voice recognition systems effectively. From a psychological perspective, since all speakers in our study were unfamiliar to participants, training might have enhanced listeners’ sensitivity to detailed speech patterns, echoing prior research indicating that familiarity improves human voice discrimination (Belin et al., 2011; Perrachione et al., 2011; Kanber et al., 2025).

The observed training effect likely resulted from participants becoming sensitive to detailed temporal differences between AI and human speech, as indicated by the TRF analyses (Fig. 2*A*), rather than from differences in long-term acoustic characteristics, as no significant differences emerged from the spectral analyses. This interpretation is plausible because modern AI speech synthesis systems are designed to mimic long-term voice statistics—learned from extensive speech data—and therefore replicate global acoustic features of human voices effectively. However, these systems may still fall short in precisely imitating moment-to-moment temporal dynamics, subtle details that participants’ auditory systems became attuned to during training, as captured by TRFs. Consistent with the dual-stream framework and hierarchical dynamic coding models (Hickok and Poeppel, 2007; Friederici, 2011), our TRF results provide a detailed temporal account of this neural differentiation. Specifically, the early differentiation observed ∼55 ms may reflect the discrimination of acoustic features and phonemic categories (Näätänen et al., 1997; Friederici, 2011). The observed left-lateralized spatial pattern at this stage aligns with the “asymmetric sampling in time” hypothesis, highlighting the left hemisphere's specialization in processing rapidly changing speech signals through short temporal integration windows (Poeppel, 2003; Oderbolz et al., 2025). Furthermore, the significant differentiation induced by short-term training at anterior and left-lateralized sites ∼55 and 210 ms may reflect adjustments in predictive coding mechanisms related to phonemic category boundaries. These processes are often associated with the dorsal stream's mapping of auditory signals to motor representations (Hickok and Poeppel, 2007; Friederici, 2011; Latinus and Taylor, 2012). At ∼455 ms, the observed differences in both posterior and anterior scalp activity may represent a transition to higher-level integrative or cross-modal processing stages (Gwilliams et al., 2025). Our findings suggest that in the increasingly challenging context of distinguishing AI-generated from human speech, listeners benefit from focusing closely on detailed speaking patterns, such as subtle variations in articulating individual speech units.

Nevertheless, although our findings provide initial insights, several questions remain open for future investigation. First, as discussed earlier, familiarity might significantly influence listeners’ sensitivity to differences between AI-generated and human speech. Our participants only briefly became familiar with test speakers during the short training and experimental sessions. A natural question is whether long-term familiarity—such as hearing AI voices cloned from well-known actors or close friends—might significantly enhance listeners’ discrimination abilities, a possibility that should be explored in future research. Second, the speech stimuli used in our study were short sentences presented without broader contexts, potentially limiting participants’ behavioral discrimination sensitivity. Subsequent research could investigate whether longer or contextually richer speech materials improve behavioral differentiation performance. Lastly, if short-term training indeed helps listeners detect acoustic differences, what exactly are those acoustic features distinguishing AI-generated from human speech? Identifying these precise features would allow targeted perceptual training to prepare listeners better for recognizing increasingly prevalent AI-generated speech. Future studies could address this question by performing detailed acoustic analyses (Teng et al., 2019)—such as comparing modulation-temporal spectra (Elliott and Theunissen, 2009)—across extensive sets of AI-generated and human speech. Collectively, our findings not only provide initial insights but also highlight further critical questions, underscoring the meaning of this work.

In conclusion, the present study reveals risks associated with the rapid development of AI speech technologies but also provides insight for developing new detection tools for AI-generated speech. Our findings reveal an important neural-behavioral dissociation: although listeners struggle behaviorally to differentiate AI-generated from human speech, brief training can induce clear neural adaptations. Future research should include longer or specialized training and additional control conditions (e.g., voices not previously encountered) to better elucidate the mechanisms of perceptual learning. These insights could ultimately inform effective strategies to enhance explicit speech discrimination skills and mitigate potential societal risks posed by increasingly sophisticated AI speech technologies.

### Statistics and reproducibility

Behavioral data were analyzed within the framework of signal detection theory (Macmillan and Creelman, 1991) in MATLAB R2022a (The MathWorks; RRID: SCR_001622) using the Palamedes toolbox 1.5.1 (RRID: SCR_006521; Prins and Kingdom, 2018). Sensitivity (*d*’) and response criterion (*c*) were calculated for each participant. These behavioral measures were then compared using a two-way repeated-measures ANOVA (rmANOVA) conducted in IBM SPSS Statistics 28.0.1.0 (RRID: SCR_016479). Significant main effects or interactions were followed by post hoc tests using paired *t* tests, with adjusted false discovery rate (FDR) correction (Benjamini and Hochberg, 1995). Significance was declared when we obtained *p* < 0.05.

Neural responses, including TRFs (0–500 ms) and frequency spectra (1–15 Hz), were compared using cluster-based permutation tests (Maris and Oostenveld, 2007) implemented with the ft_statfun_depsamplesFmultivariate function in the FieldTrip toolbox (Oostenveld et al., 2011). The significance probability was estimated via the Monte Carlo method, with 5,000 random permutations to enhance the precision of the approximation. A critical alpha level of 0.05 was applied to both temporal and spectral features.

## Data Availability

All experimental materials, including stimuli and raw data, are available in an Open Science Foundation repository (https://osf.io/d9w8a/) and have been openly accessible from the date of publication. For further requests, please contact Xiangbin Teng, xiangbinteng@cuhk.edu.hk.

## Code Availability

All custom MATLAB code, including experiment and analysis scripts, is available in the Open Science Foundation repository (https://osf.io/d9w8a/).

## Synthesis

Reviewing Editor: Luke Remage-Healey, University of Massachusetts Amherst Decisions are customarily a result of the Reviewing Editor and the peer reviewers coming together and discussing their recommendations until a consensus is reached. When revisions are invited, a fact-based synthesis statement explaining their decision and outlining what is needed to prepare a revision will be listed below. The following reviewer(s) agreed to reveal their identity: Claudia Roswandowitz.

I have received reviews from two experts in the field. both judge your work to be of interest to the field. They highlight the methodological approach being sound and the research being timely and relevant. Several points for improvement were also raised, which should be addressed in the revision of your manuscript. Regarding the last point, the SVM, please either provide a stronger justification and highlight its contribution to the findings, or remove it as suggested.

- Throughout the manuscript minor typographical issues (e.g., double punctuation, extra spaces) require correction. Also, in the subsection "Temporal response functions (TRFs) analysis" (Methods section), the very first sentence should be revised to improve clarity.

- The speech modulation spectrum methodology is missing relevant literature support (Methods section, "Acoustic analysis of stimuli" subsection).

- the manuscript lacks the interpretation of the found results related to the differences between speech types (human and AI speech) in the identified cluster of frequencies (please note that in the Results section, the authors use the terms "theta" and "alpha", which are specific to EEG analysis, for the speech modulation spectrum). Which characteristics of the speech types (prosody, syllabic structure, etc.) are associated to that frequency range and why could these differences emerge between speech types? Is there any evidence in the literature that these characteristics of the speech may influence neural perception? Please clarify.

- The results on derived TRFs would benefit from a clearer contextualization with respect to neural processing stages. In particular, the methodology does not specify which electrodes were selected for the TRF analysis (this only appears in Figure 2A, with the highlighted electrodes in green). Why was this specific brain region (and not others) considered central for acoustic auditory processing? Please clarify.

- The interpretation seems to lack a broader discussion of the significant results observed at different latencies across different cortical areas. Providing such interpretations would offer a more complete picture of the neural processes that may be involved in discriminating human and AI-generated speech.

- The rationale for including EEG spectral analyses and SVM-based decoding remains unclear. Although the power analysis is partially justified in light of the acoustic findings, it employs the cluster-based permutation approach to identify salient frequency ranges. It is not clear why the EEG power analysis was not specifically focused on testing differences between speech types within the frequency range that had already emerged as significant in the speech modulation spectrum.

- Regarding the decoding analysis, the choice of SVM is not justified, and overall the decoding results contribute little to the discussion. For these reasons, this latter analysis could be omitted without loss of clarity or impact

## Author Response

Author's Rebuttal on eNeuro eN-NWR-0300-25 24th Sep 2025 Revision Plan Summary We sincerely appreciate the editor and reviewers for their constructive feedback, which has significantly strengthened our manuscript. Below is a concise summary of the key revisions implemented to address all raised concerns:

1. Typographical &Clarity Fixes Corrected minor typographical errors (double punctuation, extra spaces) throughout the manuscript.

Revised the first sentence of the "Temporal response functions (TRFs) analysis" subsection (Methods) for clarity.

2. Literature Support for Speech Modulation Spectrum Added relevant literature to the "Acoustic analysis of stimuli" subsection (Methods) to validate the methodology's rationale and implementation.

3. Frequency Cluster Result Interpretation Replaced EEG-specific terms ("theta/alpha") with the accurate acoustic frequency range (5.4-11.7 Hz) in the Results.

Linked this frequency range to speech characteristics (syllabic rhythm, intonation, prosody) and explained AI-human speech differences in this band.

Added literature on how these speech characteristics influence neural perception.

4. TRF Analysis: Electrode Details &Rationale Explicitly specified electrodes for TRF analysis (FCz, FC1, FC2, Cz, C1, C2, CPz, CP1, CP2) in Figure 2A's caption.

Clarified the rationale for selecting these central scalp electrodes (auditory sensitivity, speech processing network association, high SNR).

5. Broader Interpretation of Latency &Scalp Distribution Results Expanded interpretation of TRF findings across latencies (55 ms, 210 ms, 455 ms) and scalp distributions.

Integrated results with theoretical frameworks (hierarchical dynamic coding, dual-stream model, predictive coding) in the Discussion.

6. Rationale for EEG Spectral Analysis Clarified the purpose of EEG spectral analysis (detect global neural differences corresponding to acoustic variations).

Explained the use of a broader 1-15 Hz range (comprehensive exploration) and confirmed supplementary analysis of the 5.4-11.7 Hz band.

7. Justification for SVM-Based Decoding Strengthened the rationale for retaining SVM decoding, highlighting its complementary role in capturing discriminative spatial neural codes (independent of temporal/spectral features).

We believe these revisions thoroughly address all reviewers' concerns while preserving the study's core contributions. Below, we provide detailed responses to each comment.

Dear Dr. Beyer, We thank the editor and two reviewers for your constructive feedback and comments on our manuscript. We have carefully considered each point raised and have made revisions accordingly. Below, we address each comment in detail. Thank you for the opportunity to resubmit our work.

Comment 1 Throughout the manuscript minor typographical issues (e.g., double punctuation, extra spaces) require correction.

Response: We appreciate the reviewer's attention to detail regarding the typographical issues in the manuscript. We have thoroughly reviewed the entire document and corrected all identified minor typographical errors, including double punctuation and extra spaces. The specific modifications are as follows:

Line 62-66: "Differentiating AI-generated speech from human speech presents a novel challenge for the human voice recognition system. Modern AI-based speech synthesis algorithms replicate critical voice features of human speech (Choi et al., 2020; Ning et al., 2019), enabling synthetic voices to frequently pass everyday "Turing tests," where listeners often fail to identify them as artificial or "deepfake" (Barrington et al., 2025; Groh et al., 2024; Mai et al., 2023; McGettigan et al., 2024; Müller et al., 2022)." Line 865-866: "The acoustic waveform corresponds to the Mandarin utterance "他很喜欢吃点心" (He enjoys eating desserts) produced by the male speaker." Line 251-253: "In the experimental sessions, for each speech condition, a two-by-two confusion matrix was constructed by treating the trials of a specific speech type as "target" and the trials of other types as "noise"." Line 255-257: "Alternatively, when one type of AI speech stimulus (e.g., fine-tuned) was treated as the "target", the other type of AI speech stimulus are discarded and the real speech stimulus was defined as "noise"." Also, in the subsection "Temporal response functions (TRFs) analysis" (Methods section), the very first sentence should be revised to improve clarity.

Response: Thank you for the valuable comment. We have revised the first sentence in the subsection "Temporal response functions (TRFs) analysis" to enhance its clarity.

Line 299-302: "The analyses in the spectral domain, such as amplitude spectral analysis, primarily quantify the distribution of signal power across different frequency components, but do not fully capture the temporal details of how neural responses track acoustic variations." Comment 2 The speech modulation spectrum methodology is missing relevant literature support (Methods section, "Acoustic analysis of stimuli" subsection).

Response: We thank the reviewer for the valuable comment. We agree that including additional literature support will help readers better understand the rationale and necessity of speech modulation spectrum analysis. We have updated the "Acoustic analysis of stimuli" subsection by adding references to both the significance of the speech modulation spectrum in speech perception and its methodological implementation.

We have added relevant references at the beginning of the "Acoustic analysis of stimuli" subsection to highlight the significance of the speech modulation spectrum in the context of speech perceptual analysis:

Line 157-162: "The speech modulation spectrum is a prominent feature in speech perceptual analysis, which is widely used to characterize the temporal structure of speech at different modulation rates (Giraud &Poeppel, 2012). Specifically, modulation spectra have been shown to capture the rhythmic properties of speech that are crucial for speech perception and neural entrainment. Evidence shows that the human auditory system is specialized for the long-term modulation spectrum and employs neural mechanisms to extract essential acoustic features from speech (Teng et al., 2021)." We have also supplemented and clarified the methodological implementation of the speech modulation spectrum analysis, adding the necessary references to support the procedure:

Line 172-176: "First, speech stimuli were filtered through a gammatone filterbank consisting of 32 logarithmically spaced frequency bands, covering a range from 50 to 8000 Hz (Ellis, 2009; Patterson et al., 1988). Subsequently, the envelope of each frequency sub-bands was obtained by applying the Hilbert transform on each band and computing the absolute values (Glasberg &Moore, 1990; Søndergaard &Majdak, 2013). " Line 177-180: "The amplitude envelope was then averaged across the 32 frequency sub-bands for each speech sentence and speaker in each speech type." Comment 3 The manuscript lacks the interpretation of the found results related to the differences between speech types (human and AI speech) in the identified cluster of frequencies (please note that in the Results section, the authors use the terms "theta" and "alpha", which are specific to EEG analysis, for the speech modulation spectrum).

Response: Thank you for your insightful comment. We appreciate your attention to the terminology and acknowledge that the use of "theta" and "alpha" in the Results section may not be the most accurate description of the speech modulation spectrum. These terms are conventionally used in EEG analysis to refer to neural oscillations, not the temporal modulations of the speech signal itself. To ensure clarity and precision, we have revised the subsection "Acoustic Differences of Speech Types in Modulation Spectra" to strictly report the identified cluster using the accurate acoustic frequency range of 5.4-11.7 Hz.

Line 431-435: "We further applied a cluster-based permutation approach to the full spectral range in order to capture potential differences in power across a broader temporal and spectral context. This analysis revealed a statistically significant cluster in the frequency range of 5.4-11.7 Hz (cluster F statistic = 351.47, p < 0.001), indicating a significant difference in modulation characteristics between AI-generated and human speech within this band." Which characteristics of the speech types (prosody, syllabic structure, etc.) are associated to that frequency range and why could these differences emerge between speech types? Response: We sincerely thank the reviewer for their thoughtful suggestion regarding the analysis of speech characteristics and their potential associations with the identified frequency range. We have provided a more detailed discussion of the differences between human and AI-generated speech in the Discussion section.

Line 529-538: "In the analysis of acoustic differences among speech types, we identified a statistically significant cluster spanning from the modulation spectrum between 5.4 and 11.7 Hz. The frequency range of 5.4-11.7 Hz captures the mid-to-high modulations in speech, representing the rapid phonemic transitions and syllabic onsets that facilitate neural tracking of the speech units (Ding et al., 2017; Varnet et al., 2017; Poeppel &Assaneo, 2020). These modulations reflect the temporal structure of natural speech, including rapid spectral changes and natural pitch contours, which are critical for the naturalness of speech. Regarding AI-generated speech, although it is high fidelity, it may fail to capture such rapid, dynamic fluctuations inherent in natural human speech (Wu et al., 2012; Paul et al., 2017; Müller et al., 2022). This absence may account for the significant differences observed in the 5.4-11.7 Hz modulation spectrum between AI-generated and human speech." Is there any evidence in the literature that these characteristics of the speech may influence neural perception? Please clarify.

Response: In the Discussion section, we have broadened our interpretation by embedding these findings in the framework of neural synchronization and have further discussed the potential influences of speech features on neural responses.

Line 538-548: "Cortical oscillations are known to be highly sensitive to the multi-timescale, quasi-rhythmic structure of speech, enabling them to dynamically track speech-related neural activity (Giraud &Poeppel, 2012). Previous research indicates that speech perception operates on distinct timescales, with neural oscillations in the theta band (~4-8 Hz) tracking syllabic rhythms (Luo &Poeppel, 2007; Poeppel, 2003), and in the alpha-beta range (8-30 Hz) tracking phonemic information (Giroud et al., 2024). These oscillations are known to synchronize with the temporal structure of speech, facilitating the parsing of linguistic units at different levels. The differences in the modulation spectrum between 5.4 and 11.7 Hz may lead to cortical phase resetting, excitatory alignment, and alterations in hierarchical information transmission (Giraud &Poeppel, 2012). These changes could, in turn, influence the neural coupling patterns and provide a potential mechanism for detecting differences between AI and human speech." Comment 4 The results on derived TRFs would benefit from a clearer contextualization with respect to neural processing stages. In particular, the methodology does not specify which electrodes were selected for the TRF analysis (this only appears in Figure 2A, with the highlighted electrodes in green). Why was this specific brain region (and not others) considered central for acoustic auditory processing? Please clarify.

Response: Thank you for highlighting the need for greater clarity in our methodological description. We appreciate your suggestion and have revised the Methods section to explicitly state the selected electrodes and the rationale for their choice. In response to this, we have revised the Methods section to explicitly clarify both the selected electrodes and the rationale for their inclusion in the TRF analysis.

Firstly, the selected electrodes used for averaging the TRF results have been explicitly specified in the Figure caption:

Line 878-880: "Temporal response function (TRF) result averaged over the selected channels (FCz, FC1, FC2, Cz, C1, C2, CPz, CP1, CP2 - The green regions on the inserted topographies)." Secondly, we have provided a clear rationale for the selection of these electrodes in the TRF analysis subsection "Neural Differentiation of Speech Types Through TRF Analysis".

Line 453-460: "We then plotted the TRF results for selected electrodes in the central scalp region (FCz, FC1, FC2, Cz, C1, C2, CPz, CP1, CP2; Fig. 2A), which were selected due to their sensitivity to auditory-related activity. The central scalp region has been associated in previous studies with a distributed network engaged in speech processing, including pathways often described as antero-ventral and postero-dorsal streams, as well as higher-level integration processes (Hickok &Poeppel, 2007; Rauschecker &Scott, 2009). Furthermore, these electrodes were selected for TRF analysis due to their relatively high signal-to-noise ratio (SNR) in EEG recordings, which is crucial for obtaining reliable and interpretable TRFs." Additionally, the central region demonstrated statistically significant activation differences across all three identified latency windows in the cluster-based permutation test when participants listened to different categories of speech stimuli. This finding provides strong empirical support for the central region's critical role in speech processing, particularly in the early stages of voice recognition and auditory perception.

Comment 5 The interpretation seems to lack a broader discussion of the significant results observed at different latencies across different cortical areas. Providing such interpretations would offer a more complete picture of the neural processes that may be involved in discriminating human and AI-generated speech.

Response: Thank you for this important and constructive comment. We have revised the "Neural Differentiation of Speech Types Through TRF Analysis" subsection in the Results section and provided a broader interpretation of the significant results observed at different latencies across scalp distributions.

Line 465-476: "The early differentiation observed around 55 ms may reflect the discrimination of acoustic features and phonemic categories (Näätänen et al., 1997; Friederici, 2011). The spatial pattern may reflect processes described in the "asymmetric sampling in time" hypothesis, which emphasizes the left hemisphere's specialization in processing rapidly changing speech signals through short temporal integration windows (Poeppel, 2003; Oderbolz et al., 2025). The short-term training induced a significant differentiation at anterior scalp sites as well as left-lateralized scalp sites around 55 ms and 210 ms, which may reflect adjustments in predictive coding mechanisms related to phonemic category boundaries during processes often associated with the dorsal stream's mapping of auditory signals to motor representations (see Discussion, Hickok &Poeppel, 2007; Friederici, 2011; Latinus &Taylor, 2012). At approximately 455 ms, we observed significant differences in both posterior and anterior scalp activity, as well as at left-lateralized scalp sites, which may reflect a later integrative or cross-modal processing stage (Gwilliams et al., 2025)." In addition, we have further elaborated on the neural processes potentially involved in distinguishing human and AI-generated speech by integrating the findings within the context of two established theoretical frameworks: the hierarchical dynamic coding of cortical language processing and the dual-stream model of speech processing in the Discussion section.

Line 569-574: "The findings in TRF analysis are consistent with models such as the hierarchical dynamic coding of cortical language processing and the dual-stream framework of speech processing (Friederici, 2011; Hickok &Poeppel, 2007). The observed scalp patterns suggest that the discrimination between human- and AI-generated speech may involve processes typically associated with the dorsal auditory stream, which is thought to potentially facilitate auditory-motor integration (Hickok &Poeppel, 2007)." Comment 6 The rationale for including EEG spectral analyses and SVM-based decoding remains unclear.

Response: Thank you for this thoughtful and important comment. EEG spectral analyses aimed to identify global neural differences between speech types across entire sentence stimuli. The rationale for including SVM-based decoding is to investigate the potential spatial coding of AI-human speech differences in EEG topographies, complementing traditional temporal and spectral analyses conducted on individual electrodes. We have clearly outlined the theoretical rationale for the EEG spectral analyses and SVM-based decoding in the manuscript:

Line 281-287: "The rationale for conducting EEG spectral analysis is grounded in the hypothesis that neural oscillations rhythmically track the temporal structure of speech (Luo &Poeppel, 2007; Poeppel &Assaneo, 2020). As illustrated in Fig. 1C, the modulation spectra of the speech stimuli display differences between AI and human speech. If the auditory brain follows this rhythmic tracking mechanism, neural responses should reflect corresponding spectral peaks in the EEG signal (Teng et al., 2020), potentially encoding differences between speech stimulus types (AI-generated vs. human-produced). To test this hypothesis, we performed amplitude spectral analysis on EEG responses." Line 501-506: "previous EEG analyses were conducted on individual EEG electrodes in the temporal and spectral domains. It is possible that AI-human speech differences could be coded across EEG electrodes (e.g., EEG topographies), reflecting spatial neural codes of AI-human speech information (King et al., 2016). To examine this possibility, we conducted a decoding analysis, classifying speech types at each time point using data from all 61 electrodes and leveraging the topographical information across electrodes." Although the power analysis is partially justified in light of the acoustic findings, it employs the cluster-based permutation approach to identify salient frequency ranges. It is not clear why the EEG power analysis was not specifically focused on testing differences between speech types within the frequency range that had already emerged as significant in the speech modulation spectrum.

Response: With regard to the reviewer's question about why the EEG power analysis was not specifically restricted to the frequency range (5.4-11.7 Hz) that showed significant differences in the speech modulation spectrum, we clarify that the broader 1-15 Hz range was selected to allow for a more comprehensive examination of potential neural differences across a wider temporal and spectral context. Although the speech modulation spectrum highlighted a specific range of interest, we aimed to explore whether additional frequency bands might also contribute to the differentiation between human and AI-generated speech. To ensure statistical rigor in this exploratory analysis, we employed the cluster-based permutation approach, which effectively accounts for multiple comparisons and spatial dependencies inherent in EEG data. We have updated the manuscript to clarify this rationale:

Line 486-489: "The speech modulation spectrum analysis revealed a statistically significant cluster within the frequency range of 5.4-11.7 Hz. To capture potential power differences across a broader temporal and spectral context, we derived the amplitude spectra of neural responses within the frequency range of 1 to 15 Hz and analyzed whether these spectra differed across speech types." Line 294-297: "In order to achieve a more comprehensive examination of potential neural differences across a broader temporal and spectral context, we analyzed EEG power both within the specific 5.4-11.7 Hz frequency band and across the wider 1-15 Hz range." We also directly examined the EEG power in the 5.4-11.7 Hz frequency band. The results did not reveal any significant differences.

Line 494-497: "We also examined the EEG power specifically within the 5.4-11.7 Hz band in both sessions, and the cluster-based permutation tests indicated no significant differences between conditions (a positive cluster was identified in Session 1: cluster F-statistic = 48.9 , p = 0.589; no positive cluster was identified in Session 2). This finding aligns with the results of the broader spectral analysis." Comment 7 Regarding the decoding analysis, the choice of SVM is not justified, and overall the decoding results contribute little to the discussion. For these reasons, this latter analysis could be omitted without loss of clarity or impact.

Response: We appreciate the reviewer's comment regarding the decoding analysis. We agree that the justification for the decoding analysis could be more robustly articulated, and we appreciate the opportunity to clarify its contribution to our study. The decoding results provide a complementary analytical perspective to the temporal response function (TRF) analysis and the EEG spectral analyse. Specifically, the three distinct methods-TRF analysis, EEG spectral analysis, and EEG decoding-each capture distinct aspects of neural processing, as we have elaborated in the manuscript:

Line 241-245: "(2) at the neural level, TRFs would reveal temporal neural tracking differences post-training (Teng et al., 2017, 2019, 2024); (3) EEG spectral analyses would identify global neural differences between speech types across entire sentence stimuli (Teng et al., 2017, 2020); and (4) EEG decoding analyses would reveal whether spatial EEG patterns provide discriminative spatial neural codes differentiating human from AI-generated speech (Cichy et al., 2014)." To conclude: * TRF analysis reveals temporal dynamics of stimulus-tracking, but does not assess whether the spatial configuration of activity across the scalp discriminates speech types. * Spectral analysis identifies global power modulations, yet cannot determine whether speech categories are linearly separable based on multivariate spatial patterns. * Decoding analysis tests whether the topographic distribution of neural activity carries discriminative information independent of temporal or spectral amplitude. This is crucial because different neural sources could produce similar frequency responses yet exhibit distinct spatial signatures, which only multivariate decoding can detect.

Furthermore, EEG decoding analyses computed at each time point serve as a complementary approach to the significant findings from TRF analysis. This method enables the investigation of the spatial dimension of neural coding, thereby extending the temporal insights provided by TRFs and contributing to a more comprehensive understanding of the neural mechanisms underlying speech perception. We have included this rationale in the Results section, within the subsection "Limited EEG Spatial Differentiation of Human and AI Speech".

Line 499-504: "In the TRFs analysis, we revealed distinct post-training temporal neural tracking patterns, indicating differences in how neural activity follows the acoustic features of human versus AI-generated speech over time. However, previous EEG analyses were conducted on individual EEG electrodes in the temporal and spectral domains. It is possible that AI-human speech differences could be coded across EEG electrodes (e.g., EEG topographies), reflecting spatial neural codes of AI-human speech information (King et al., 2016)." We believe that retaining the decoding analysis preserves a multi-dimensional and more comprehensive view of the neural mechanisms involved in speech perception, thereby contributing positively to the overall clarity and impact of the study. However, to enhance the manuscript's clarity and focus, we have removed the decoding analysis figure (Fig. 2C) from the revised version. The decoding results remain included in the Methods and Result sections to retain a comprehensive view of the neural mechanisms involved in speech perception.
